# Vertical differences in carbon metabolic diversity and dominant flora of soil bacterial communities in farmlands

**DOI:** 10.1038/s41598-024-60142-2

**Published:** 2024-04-24

**Authors:** Bufan Zheng, Zhipeng Xiao, Jiaqi Liu, Yi Zhu, Kaifeng Shuai, Xiaye Chen, Yongjun Liu, Ruiwen Hu, Guangjue Peng, Junlin Li, Yichao Hu, Zan Su, Ming Fang, Juan Li

**Affiliations:** 1 Agronomy College, Hunan Agricultural University, Changsha, 410128 China; 2 Hunan Tobacco Monopoly Bureau, Changsha, 410004 China; 3 Hubei Tobacco Industry Co., Ltd., Wuhan, 430040 China; 4 Guangxi Tobacco Industry Co., Ltd., Nanning, 530001 China

**Keywords:** Soil microbiology, Agroecology

## Abstract

The carbon cycle in soil is significantly influenced by soil microbes. To investigate the vertical distribution of the dominant groups in agricultural soil and the carbon metabolic diversity of soil bacteria, 45 soil samples from the 0 ~ 50 cm soil layer in Hunan tobacco–rice multiple cropping farmland were collected in November 2017, and the carbon diversity of the soil bacterial community, bacterial community composition and soil physical and chemical properties were determined. The results showed that the carbon metabolic capabilities and functional diversity of the soil bacterial community decreased with depth. The three most widely used carbon sources for soil bacteria were carbohydrates, amino acids, and polymers. The dominant bacterial groups in surface soil (such as Chloroflexi, Acidobacteriota, and Bacteroidota) were significantly positively correlated with the carbon metabolism intensity. The alkali-hydrolysable nitrogen content, soil bulk density and carbon–nitrogen ratio were the key soil factors driving the differences in carbon metabolism of the soil bacterial communities in the different soil layers.

## Introduction

Land serves as an indispensable material foundation and resource carrier for human survival and social development and provides important ecological and economic benefits^1^. Soil organic carbon is one of the main components of soil, and it is one of the important factors for characterizing the soil quality and maintaining the productivity of terrestrial ecosystems^2^. The carbon cycle is an important part of the biogeochemical cycle. The spatial heterogeneity in soil, development time, climatic zone, vegetation, hydrology and other comprehensive environmental factors directly or indirectly affect the carbon cycle in soil^3^.

Carbon and nitrogen metabolism is a necessary physiological activity for the survival and growth of various organisms. In farmland ecosystems, soil organic carbon is mainly derived from crop litter, tillage measures and crop root exudates^4^. The conversion of plants into soil organic carbon, through decomposition and eventual stabilization, is mainly achieved by microorganisms. Under the action of soil microorganisms, most of the organic matter is decomposed into CO_2_ and released back into the atmosphere. A small amount of organic matter is difficult to decompose and eventually persists in the soil in the form of stable organic matter (mainly humus). A high carbon-to-nitrogen ratio (C/N) may be more beneficial for SOC sequestration^5^, but litter input generally favours soil nitrogen retention, reduces soil C/N, and increases microbial activity^6^, which is a major factor in multiple SOC changes due to increased nitrogen. The diverse conditions found within soil particles encourage the growth of different types and amounts of soil microorganisms, and changes in these factors can impact the soil quality^7,8^. Microorganisms can adapt to different growth environments by finely regulating the carbon and nitrogen metabolism balance^9,10^. The spread of soil microorganisms can be influenced by environmental conditions. For example, studies have indicated that the pH, soil water conditions, and organic matter dissolution are the key determinants influencing microbial biogeographic patterns^11^, while soil nutrients such as total nitrogen and total phosphorus exert considerable impacts on the soil microbial abundance^12^. Bolan et al.^13^ reported that most dissolved organic carbon is consumed during soil microbial activities and can provide suspended organic carbon particles for adsorption during soil aggregate formation, which plays a major role in the initial aggregate composition process. Soil microorganisms constitute the driving force of material and energy cycling in soil, and their survival and reproduction are mainly provided by organic carbon in soil^14^.

Soil microorganisms participate in the formation and decomposition of humus in soil and the transformation and cycling of soil nutrients, and they promote the energy flow and material cycle in terrestrial ecosystems^15–17^. They are important indicators for evaluating agricultural practices^18^, are often regarded as sensors of agricultural ecosystems, and fulfil an important role in ecosystem functions through interactions with the surrounding environment^19^. Numerous researchers have investigated bacterial communities in surface and subsurface soils, and while considerable differences in the bacterial community structure between the two types of soils have been reported^20,21^, the soil microbial communities in both soils affect the soil material cycle and physiological ecology of plants^22^.

However, most studies on soil bacterial carbon metabolism have focused on near-surface soil (0 ~ 15 cm)^23,24^, with little data on the vertical functional organization of soil bacterial populations. Therefore, variations in the carbon utilization capacity and organization of microbial communities, as well as the contribution of the deep soil layer to the soil carbon cycle, may be represented by the ability of soil bacterial communities to metabolize carbon in the vertical dimension.

Therefore, we collected soil profiles from typical tobacco–rice multiple cropping areas (Changsha, Hengyang, and Chenzhou) in Hunan Province, studied the relationships between soil bacterial carbon source metabolic activity and different soil chemical and physical properties at various depths through the Biolog-ECO test method and 16S sequencing analysis, and analysed the influence of environmental factors on metabolic activity. The objective of this study was to define the vertical distribution sequence of the soil bacterial capacity for carbon metabolism and to better understand how variations in local soil characteristics affect the soil carbon metabolism functionality of different soil layers and impact the capacity of soil microbial communities to metabolize carbon, thereby providing a systematic basis for understanding soil quality changes and fostering sustainable utilization of tobacco–rice multiple cropping fields in Hunan.

## Materials and methods

### Location of the research area

The research region is located in the southeastern area of Hunan Province, China, which belongs to the subtropical monsoon humid climate zone. The average temperature generally ranges from 16 to 19 °C, the annual rainfall ranges from 1200 to 1700 mm, and the frost-free period is 253–311 days. The soil exhibits a loam texture.

### Sampling

In November 2017 (after the rice harvest), three typical fields with approximately 10 years of tobacco and rice planting were selected in the tobacco–rice multiple cropping areas of Baiyun village (25°46′31′′N, 112°40′30′′E), Renyi town, Guiyang County, Chenzhou city (26°40′17′′N, 112°58′18′′E), Yanzhong village, Mashui town, Leiyang city, Hengyang city (26°40′17′′N, 112°58′18′′E) and Binghe village, Guandu town, Liuyang city, Changsha city (28°20′31′′N, 113°55′34′′E). We utilized a soil column sampler of a specific design, measuring 50 cm in length and 7.5 cm in diameter, to successfully collect undisturbed soil columns, each 50 cm long, at six randomly chosen locations within each representative field. We divided each obtained soil column into five layers at intervals of 10 cm. Subsequently, all the same soil layer samples collected at the six locations within the same field (500 m^2^ each) were mixed, ensuring that the sample represented the overall characteristics of the particular soil layer in the field. Next, each combined soil sample was evenly divided into three portions. Consequently, for each typical field and its corresponding soil layers, we obtained three independent soil samples. By this method, we acquired a total of 45 soil samples across all the fields (i.e., 5 soil layers per field * 3 samples per soil layer * 3 fields = 45 soil samples).

### Determination of the soil physical and chemical properties

After the soil samples were dried, the soil physicochemical properties were determined according to a previous protocol^25^: the soil pH was measured using a potentiometric method. Soil organic carbon (SOC) was determined using a total organic carbon analyzer (Vario TOC Cube, Elementar Analysensysteme GmbH, Germany). The total nitrogen (TN) and available nitrogen contents were rapidly determined using an automated Kjeldahl apparatus. After the SOC and total nitrogen data were obtained separately, the carbon-to-nitrogen ratio (CNR) was calculated. The soil moisture content was assessed via oven-drying, the soil bulk density was measured via the core cutter method, and the soil porosity was indirectly derived via density calculations.

### Determination of the soil capacity for utilizing different carbon sources

The characteristics of soil microbial carbon source consumption were examined with the Biolog-ECO method^26^. Representative soil samples were collected from the target locations and transported to the laboratory for natural drying and grinding. Subsequently, an appropriate amount of soil was mixed with sterile water at a ratio of 1:50 (g), and a microbial suspension was prepared by shaking at 200 rpm for 30 min to ensure a uniform distribution of microorganisms. The suspension concentration was adjusted to 0.15 OD at a wavelength of 600 nm to guarantee an appropriate microbial cell density. Thereafter, equal volumes of the microbial suspension were inoculated into each well of a Biolog microplate. The inoculated microplates were placed in a thermostatic incubator maintained at 28 °C, where continuous cultivation occurred for 10 days with predetermined intervals of 24 h between measurements. During cultivation, the microorganisms utilized various carbon sources within the microplate wells, undergoing metabolic activities that produced reduced products that reacted with colour-developing agents, resulting in colour changes. The absorbance values were measured at 590 and 750 nm using an ELx808TM Microplate Reader (brand: Baiteng, United States) at 0, 24, 48, and 240 h with 24-h intervals. The average well colour development (AWCD), Shannon species diversity index (H), and Simpson dominance index (D)^27^ were then calculated during the course of the experiment as follows:

1 $$AWCD = \sum {(C_{i} - {\text{R}})} /31$$

where C*i* denotes the difference between the absorbance values of the *i*th carbon source hole at 590 and 750 nm, and R denotes the absorbance of the control hole.

2 $$Shannon=-\sum {P}_{i} \times \mathrm{ ln}\left({P}_{i}\right)$$

3 $$Simpson=1-\sum {{P}_{i}}^{2}$$

where P*i* is the ratio of the relative absorbance of the *i*_th_ carbon source hole to the sum of the relative absorbance of the whole plate.

### DNA extraction, PCR amplification, MiSeq sequencing, and data processing

DNA was isolated from 45 soil samples using an Omega bacterial DNA kit (Omega Genetics), and Thermo NanoDrop One (Thermo Fisher Scientific) was used to measure the DNA purity and concentration. With the use of genomic DNA as a template, the V4 region of the 16S rRNA gene was amplified by independent PCR using the primer sets 515F (3'-GTGCCAGCMGCCGCGGTAA-5') and 806R (3'-GGACTACHVGGGTWTCTAAT-5'). The resulting amplicon library was subjected to PE6000 sequencing on the Illumina Nova 250 platform^28^. Following initial collection of the sequencing data, we first removed the primers using the Cutadapt program (https://github.com/marcelm/cutadapt/). Then, following the quality control parameters, we acquired paired-end clean reads. Next, OTU clustering was conducted using UPARSE at a 97% similarity criterion, and FLASH was used to combine forward and reverse sequences^29^. Finally, taxonomic classification assignment was performed by the ribosomal database item classifier, and the minimum confidence was set to 50%. To account for different sequencing depths, each sample was resampled to 42,303 sequences, which were then divided into 18,190 OTUs. A publicly available galaxy analysis system (http://mem.rcees.ac.cn:8080/) was used for the analyses^30^.

### Statistical analysis

The data analytics and tabulation program used was Excel 2019, and the mapping software employed was Origin 2018. To identify significant differences between treatments, SPSS 22.0 (SPSS Inc., Chicago, Illinois, USA) was used with analysis of variance (ANOVA) and least significant difference (LSD) approaches. Differences were considered statistically significant at *p* < 0.05. To measure the difference, Duncan's multiple range approach was applied. SigmaPlot 12.0 (Systat Software, Inc., San Jose, CA, USA) was used to create the figures. The primary discriminant categories in the bacterial population were investigated using linear discriminant analysis (LDA) and linear effect size (LEfSe) approaches during the treatment (LDA score > 3.5, *p* < 0.05). Spearman's correlation was used to assess the associations between the bacterial populations in the soil samples and other elements.

## Results

### Ability of the bacterial communities in the different soil layers to utilize carbon sources

**Figure 1:**
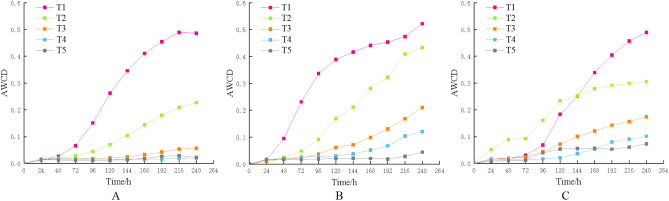
AWCD values of the bacterial communities in the different soil layers (A. Chenzhou; B. Hengyang; C. Changsha). *Note* T1: 0 to 10 cm soil layer; T2: T2: 10 to 20 cm soil layer; T3: 20 to 30 cm soil layer; T4: 30 to 40 cm soil layer; T5: 40 to 50 cm soil layer; same below.

AWCD may be used to determine the capacity of a soil microbial community to metabolize different carbon sources. Figure 1 shows the overall performance of the AWCD value of soil bacterial carbon source consumption in the different Hunan tobacco–rice multiple cropping fields. The AWCD value decreased with increasing soil depth, indicating that bacteria in the topsoil layer could better utilize carbon sources than those in the deep soil layers. Notably, the carbon metabolism capacity of the soil bacterial community in Chenzhou (Fig. 1A) sharply decreased in layer T2 (10 ~ 20 cm soil layer). However, in Hengyang (Fig. 1B) and Changsha (Fig. 1C), the soil bacterial community potential for carbon metabolism decreased to an extremely poor level in layer T4 (30 ~ 40 cm soil layer).

### Analysis of the carbon metabolism functional diversity of the bacterial communities in the different soil layers

In line with the shifting trend of the AWCD value curve, we selected an AWCD value of 240 h to investigate the composition of the soil bacterial population. The outcomes are listed in Table 1. With increasing soil layer depth, the Shannon and Simpson index values at the three locations decreased. There was a significant difference in the Shannon and Simpson index values between the topsoil (0 ~ 20 cm) and subsoil (20 ~ 50 cm) layers in Chenzhou and Hengyang, while the Shannon and Simpson index values in Changsha decreased with soil depth. Moreover, there was no significant difference between the different soil layers.

**Table 1:** Diversity index values of the bacterial communities in the different soil layers in Hunan.

Tillage	Chenzhou			Hengyang			Changsha		
	AWCD	Shannon	Simpson	AWCD	Shannon	Simpson	AWCD	Shannon	Simpson
T1	0.49a	3.13a	0.95a	0.52a	3.23a	0.96a	0.49a	2.94a	0.94a
T2	0.23b	2.94a	0.94a	0.43a	3.11b	0.95a	0.31b	2.95a	0.94a
T3	0.06b	2.43b	0.86b	0.21b	2.84b	0.92b	0.17c	2.82a	0.93a
T4	0.02b	2.62b	0.91a	0.12bc	2.62bc	0.91c	0.10c	2.50a	0.89a
T5	0.02b	2.60b	0.90ab	0.04c	2.45c	0.89d	0.07c	2.47a	0.89a

Significant differences in the same data column are marked with different letters (*p* < 0.05).

### Intensity of the use of various carbon sources by the bacterial populations in the different soil layers

**Figure 2:**
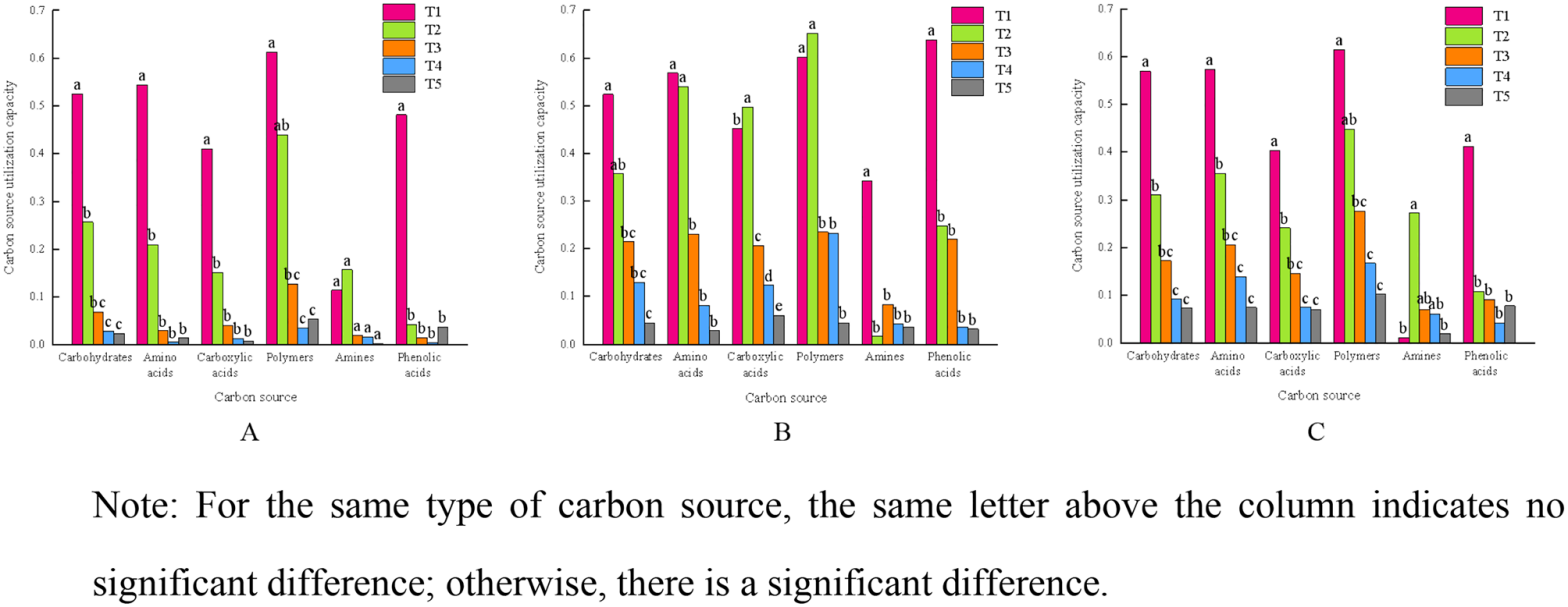
Ability of the bacterial communities in the different soil layers to utilize the various carbon sources (A. Chenzhou; B. Hengyang; C. Changsha).

The 31 carbon sources on the Biolog ECO microplate can be categorized into six types, namely, carbohydrates (10), amino acids (6), carboxylic acids (7), polymers (4), amines (2) and phenolic acids (2). The ability of soil microorganisms cultured for 240 h to utilize the different types of carbon sources was analysed, and the results are shown in Fig. 2. The ability of bacteria to utilize the six distinct carbon source types across the multiple soil layers generally decreased with soil layer depth.

In the Chenzhou area (Fig. 2A), compared to the other soil layers, T1 exhibited a much greater level of consumption of carbohydrates, amino acids, carboxylic acids, and phenolic acids. Greater utilization of polymers was observed in T1 than in T3, T4, or T5, and significantly higher polymer utilization was observed in T2 than in T3 and T5. There were no notable differences in the capacity of bacteria to use amines among the five soil layers.

In the Hengyang area (Fig. 2B), compared to layers T3, T4, and T5, layer T1 attained a much greater capacity for bacterial glucose utilization. The bacteria in the T1 and T2 soil layers used amino acids to a far greater extent than those in the remaining three soil layers. The ability to use phenolic acids was significantly higher in the T1 layer than in the other soil layers. The ability of bacteria to use carboxylic acids and polymers was significantly greater in the T1 and T2 layers than in the other soil layers, and layer T1 exhibited a much higher capacity for amine utilization.

The T1 layer in Changsha (Fig. 2C) exhibited a significantly greater capacity than the other layers for the utilization of amino acids, carboxylic acids, and carbohydrates. Layer T1 also indicated a substantially higher capacity for polymer consumption than layers T3, T4, and T5. Compared to the other soil layers, T1 exhibited a much higher capacity for phenolic acid use.

### Principal component analysis of bacterial carbon source metabolism in the different soil layers

**Figure 3:**
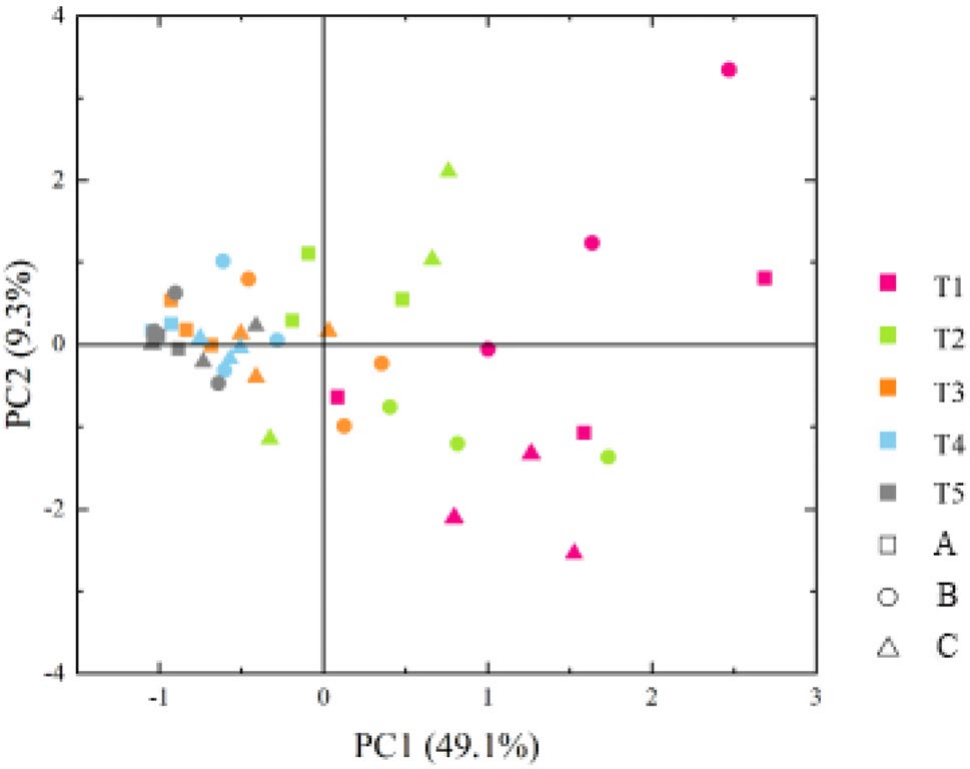
Analysis of the principal components of bacterial carbon source metabolism across the various soil layers (A. Chenzhou; B. Hengyang; C. Changsha).

Figure 3 shows that PC1 and PC2 could explain 49.1% and 9.3% of the total variance, respectively, and that the combined contribution to the variance reached 58.4%. While the separation between CZ, HY and CS was not obvious on the PC1 and PC2 axes, there was a clear distinction between the various soil strata. All the soil layer T1 samples and the majority of the layer T2 samples had positive PC1 values. Soil layer T3 showed the opposite pattern to that of layer T2, while layers T4 and T5 exhibited entirely negative PC1 values. The five soil layers were spread along the positive and negative axes of PC2, with the deeper soil layers indicating a tendency to cluster closer to the zero value of the PC2 axis. As a result, it was clear that while there were differences between the three farming regions of Chenzhou, Hengyang, and Changsha in terms of the capacity of the soil bacterial communities to utilize carbon sources, these differences were not as significant as the differences between the various soil layers.

In principal component analysis, the load values of the 31 carbon sources (Table S1) could reflect the closeness of the relationships between these carbon sources and the principal component. The higher the absolute value of the load value was, the closer the relationship between the carbon source and the principal component. There were 20 carbon sources with an absolute load factor of the main component PC1 greater than 0.6. These sources included five carbohydrates, four amino acids, five carboxylic acids, four polymers, and two phenolic acids. There were 10 carbon sources with absolute values above 0.8, including two carbohydrates (D-xylose and D-mannitol), three amino acids (L-asparagine, L-phenylalanine and L-serine), 2 carboxylic acids (γ-hydroxybutyric acid and methyl pyruvate), two polymers (Tween 40 and Tween 80), and one phenolic acid (4-hydroxybenzoic acid). Only phenylethylamine exhibited an absolute load value of the primary component PC2 greater than 0.6, but there were no carbon sources with a load value greater than 0.8. A thorough investigation revealed that the vertical characteristics of the carbon metabolic functions of the soil bacterial populations in the tobacco–rice multiple cropping fields in Hunan were closely related to carbohydrates, amino acids, carboxylic acids, and polymers.

### Differential vertical distributions of the dominant bacterial groups among the soil layers

**Figure 4:**
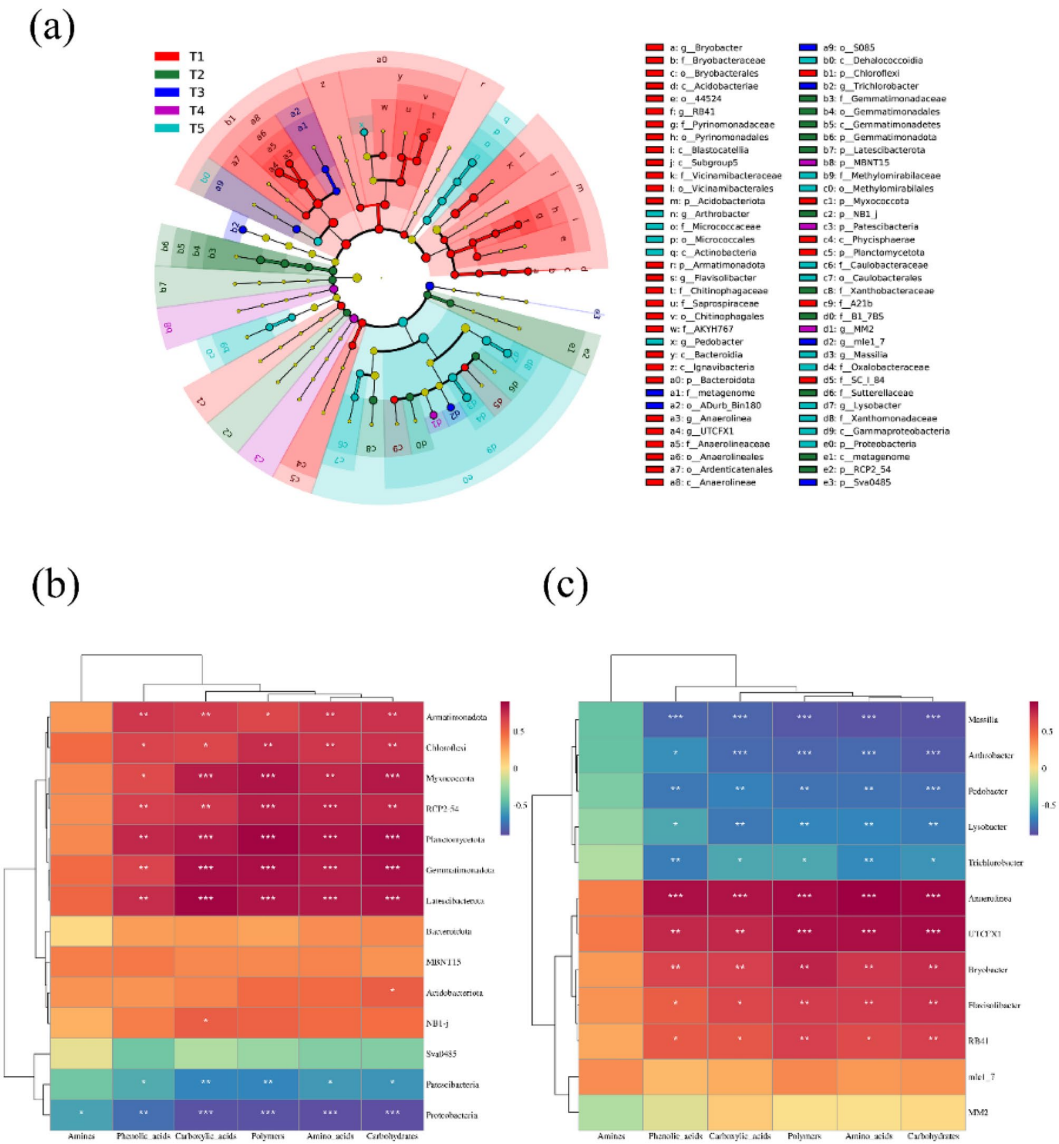
(**a**) Analysis of the soil microbial communities in the Hunan tobacco–rice-growing fields using LEfSe. (**b**) and (**c**) Spearman’s correlations between specific bacterial phyla and genera and the carbon source utilization intensity. The impact size of linear discriminant analysis (LDA) on the bacterial communities in the various soil layers (LEfSe) is shown (LDA > 3.5, *p* < 0.05). Significant (red, green, blue, purple, and light blue) and nonsignificant (yellow) discriminant classification nodes are represented by de different colours. From the centre outwards, each ring shows a taxonomic hierarchy: border, phylum, class, order, family, and genus. Hue denotes the Spearman correlation coefficient. The grid asterisks indicate high Spearman’s correlations: *p < 0.05; **p < 0.01; ***p < 0.001.

From the phylum level to the genus level, effect size analysis using linear discriminant analysis was applied, and the bacterial community groups exhibiting differences in each soil layer of the tobacco–rice multiple cropping fields were compared. The results (Fig. 4a, Table S2) showed that 70 taxa demonstrated significant differences between the five soil layers. There were considerably more bacteria exhibiting differential distributions in layer T1 than in the other soil layers for six phyla (Acidobacteriota, 14.95%; Armatimonadota, 0.99%; Bacteroidota, 10.22%; Chloroflexi, 30.79%; Myxococcota, 1.65%; and Planctomycetota, 3.01%) and five genera (*Bryobacter,* 0.83%; *RB41*, 0.89%; *Flavisolibacter*, 1.34%; *Anaerolinea*, 0.81%; and *UTCFX1*, 13.56%). The relative abundances of four phyla (Gemmatimonadota, 2.61%; Latescibacterota, 1.49%; NB1_j, 0.68%; and RCP2_54, 1.01%) were considerably higher in layer T2 than in the other layers. Compared to the other soil layers, T3 exhibited considerably higher relative abundances of microorganisms in one phylum (Sva0485, 6.16%) and two genera (*Trichlorobacter*, 1.05%; and *mle1_7*, 0.75%). Two phyla (MBNT15, 2.83%; and Patescibacteria, 4.77%) and one genus (*MM2*, 0.94%) attained considerably greater relative abundances in the T4 soil layer than in the other soil layers. Compared with the other soil layers, the T5 soil layer indicated a significantly higher relative abundance of one phylum (Proteobacteria, 37.63%) and four genera (*Arthrobacter*, 1.81%; *Pedobacter*, 0.70%; *Massilia*, 6.22%; and *Lysobacter*, 22.64%). Overall, the distinctive bacterial communities encountered in each soil layer may be one of the elements causing variations in soil microbial carbon metabolism.

The relationships between the distinct bacterial groups in each soil stratum and their carbon source use levels are shown in Fig. 4b and c. At the phylum level, the relative abundance in the topsoil layer (0–20 cm) was notably greater than that in the subsoil layers. In addition to amines, the degree of metabolism of carbon sources was significantly positively related to Armatimonadota, Chloroflexi, Myxococcota, Planctomycetota, Gemmatimonadota, Latescibacterota, and RCP2_54. The relationship between Acidobacteriota and carboxylic acids was statistically significant, as was the relationship between NB1_j and carbohydrates. There was a substantial negative correlation between five carbon sources, with amines as the exception, and Patescibacteria, and the relative abundance of this group in the subsoil layers was notably greater than that in the topsoil layer. Proteobacteria exhibited significant negative correlations of different degrees with the degree of metabolism of the six carbon sources. At the genus level, there was a significant difference between the relative abundance levels of *Bryobacter*, *RB41*, *Flavisolibacter*, *Anaerolinea*, and *UTCFX1* in the topsoil and subsoil layers, and there was a significant positive association between the metabolic degree of five carbon types, with the exception of amines. There was a substantial negative correlation between the metabolism of five carbon types, with the exception of amines, and the relative abundances of *Trichlorobacter*, *Arthrobacter*, *Pedobacter*, *Massilia*, and *Lysobacter* in the subsoil layers, which were significantly greater than those in the topsoil layer.

### Soil factors driving carbon metabolism of the bacterial populations in the different soil layers

**Figure 5:**
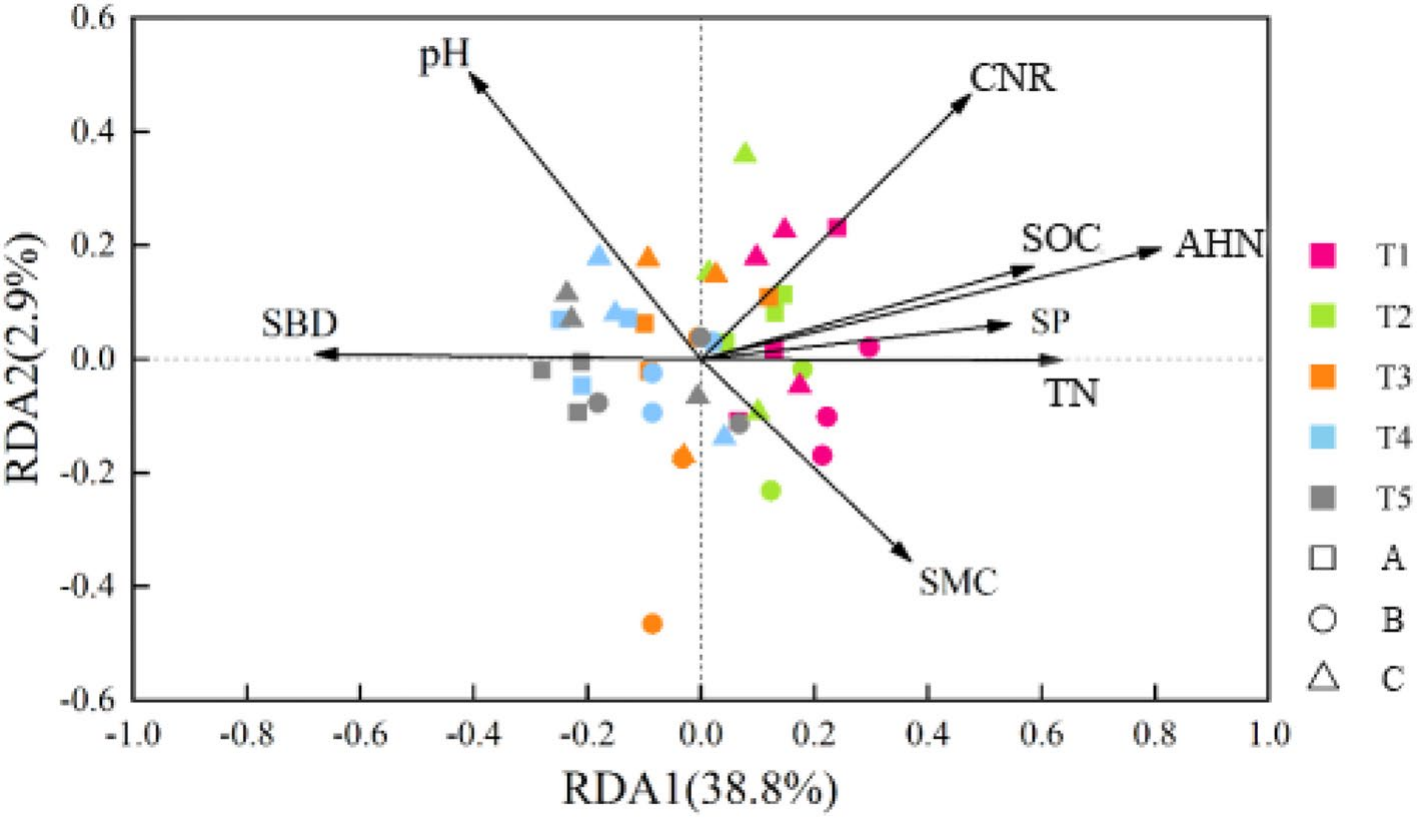
Redundancy Analysis of the Relationships between Soil Environmental Factors and the Carbon Source Utilization Capacity of Bacterial Communities (A. Chenzhou, B. Hengyang, C. Changsha, pH. Soil pH value, SP. Soil Porosity, SMC. Soil Moisture Content, TN. Total Nitrogen, AHN. Alkali-hydrolysable Nitrogen, and CNR. Carbon-to-Nitrogen Ratio, SOC. Soil organic carbon, SBD. Soil Bulk Density).

Table S3 provides the test results for the physical and chemical characteristics of the soil layers in the different agricultural areas. Data on the level of carbon source exploitation and the physical and chemical compositions of the soil samples were subjected to redundancy analysis. Table S4 and Fig. 5 show the correlation between the amount of carbon actively metabolized by the soil bacterial populations and the physical and chemical characteristics of the different soil layers. The two axes of RDA1 and RDA2 cumulatively accounted for 41.7% of all the variables. The first axis of RDA explained 38.8% of the total variables, the second axis of RDA explained 2.9% of the total variables, and the correlation analysis results were key to the RDA1 axis. The soil layers, from shallow to deep, ranged from positive to negative on the RDA1 axis. Among the soil properties, SP, SMC, SOC, TN, AHN, and CNR were closely associated with the RDA1 axis, while SBD and pH were not. The impacts of the pH and SBD on soil microbial carbon metabolism were generally inversely proportional to the soil depth. The impacts of SP, SMC, SOC, TN, AHN, and CNR on soil microbial carbon metabolism increased with decreasing soil depth.

## Discussion

The soil depth may cause changes in many soil environmental factors. For instance, the changes in the quantity of soil nutrients at various depths are indirectly influenced by the accessibility of soil moisture^31^, which consequently influences the diversity and community structure of soil microorganisms^32^. Numerous studies have demonstrated that the community framework and operational structure of soil microorganisms change in relation to the location^33^, climate^34^, soil properties^35^, and tillage technique^36,37^.

The findings of this study demonstrated that in the Hunan tobacco–rice multiple cropping fields, the ability of soil bacterial communities to metabolize carbon decreased with increasing soil depth and that both the range of species and abundance of the soil bacterial community generally decreased with increasing soil depth. Similar to the findings of Erhunmwunse et al.^38^, the bacterial diversity peaked at 10 cm from the top of the soil profile. Through LEfSe analysis, we found that the bacterial communities with large differences in soil composition in each layer were correlated with the intensity of carbon source metabolism. Notably, the unique bacterial communities in the topsoil layer can boost carbon metabolism, which is favourable for increasing the degradation and transformation of organic carbon, thereby creating favourable material and energy cycles^39^. Specific subsoil bacterial populations may inhibit carbon metabolism in soil, limiting the rate of transformation and degradation of soil organic carbon and thus preventing the accumulation of organic carbon^40^.

Our determined physical and chemical characteristics revealed that with increasing soil depth, the soil bulk density, porosity, water content, organic matter content, and total nitrogen content decreased. The findings of a correlation study showed that there was a significant relationship between soil microorganisms and soil physical and chemical characteristics. The microbial community composition changes with soil bulk density. An increased bulk soil density results in decreased soil aeration and porosity, which is generally unfavourable for the development of soil microbial communities^41^. An increase in organic input is beneficial for increasing the microbial diversity in soil^42^. This occurs because soil organic matter itself contains a large amount of carbon and participates in the synthesis of various carbon sources for microbial utilization^43^. Increased microbial diversity may accelerate the turnover rate of nitrogen in soil^44^, which suggests that higher microbial diversity results in the provision of more nutrients for promoting plant growth^45^. Therefore, a reduction in soil physical and chemical qualities results in a decrease in soil bacterial diversity and abundance, as well as a reduction in the capacity to metabolize carbon, all of which affect the living conditions of soil microorganisms in the deeper soil layers^46^.

We believe that this may be due to the effects of tillage. In long-term shallow rotary tillage, soil nutrients are exposed at the surface, thereby bringing the bottom layer of fertile soil to the surface^47^, resulting in an uneven soil nutrient distribution and low microbial enrichment in deep soil. In the case of perennial rotary tillage, the depth of the local soil plough layer is shallow, with severe soil compaction, which hinders the development of the crop root system. The roots of the two local crops are mostly concentrated in the 0 ~ 20 cm soil layer^48^, and root exudates and stubble residues provide a stable growth environment and various nutrients for the surrounding soil microorganisms^49^. Finally, a progressive deterioration in the soil quality is caused by the vicious cycle of deteriorating soil physical and chemical qualities and soil bacterial carbon metabolism. To obtain a practical way to enhance the physical and chemical characteristics of deep soil and enhance the capacity of deep soil bacterial communities to metabolize carbon, it is therefore necessary to analyse the changes in the structure of microbial communities by optimizing tillage measures^50^ and to thoroughly investigate the coupling mechanism between the community structure and function^51^.

## Conclusion

According to our findings, the capacity of bacterial populations to metabolize carbon decreased from the surface soil layer to the deep soil layers, 0–50 cm soil, in tobacco–rice multiple cropping fields in Hunan. The most utilized carbon sources were carbohydrates, amino acids, and polymers. The vertical properties of soil bacterial community carbon metabolism were closely correlated with carbohydrates, amino acids, carboxylic acids, and polymers. The dominant bacterial groups in the topsoil (such as Chloroflexi, Acidobacteriota and Bacteroidota) and subsoil (such as Proteobacteria and Patescibacteria) layers were significantly positively and negatively correlated, respectively, with the carbon metabolism intensity. The key soil environmental parameters that influenced the variations in carbon metabolism of the bacterial communities in the different soil strata were SP, SBD, SOC, AHN, and CNR.

### Supplementary Information

Supplementary Information.

## Data Availability

The data that support the findings of this study are available from the corresponding author upon reasonable request. Raw 16S rRNA gene sequences were submitted to the National Center for Biotechnology Information (NCBI) Sequence Read Archive (SRA) under accession number PRJNA833260.
