# Supplementary material for: Vertical differences in carbon metabolic diversity and dominant flora of soil bacterial communities in farmlands
Source: Sci Rep. 2024 Apr 24;14:9445. doi: 10.1038/s41598-024-60142-2 (PMC11043072; doi:10.1038/s41598-024-60142-2)
Supplement: Supplementary file 1 — Supplementary Information. [file 41598_2024_60142_MOESM1_ESM.docx]

Table S1 Principal component loading values of 31 carbon sources

| Carbon source | Carbon source composition | PC1 | PC2 |
| --- | --- | --- | --- |
| Carbohydrate | D-Galactonic Acid γ-Lactone | 0.475 | -0.345 |
|  | β-Methyl-D-Glucoside | 0.585 | -0.255 |
|  | D-Cellobiose | 0.762 | 0.258 |
|  | α-D-Lactose | 0.451 | 0.123 |
|  | i-Erythritol | 0.750 | -0.085 |
|  | α-D-Glucose-1-Phosphate | 0.576 | 0.213 |
|  | D-Xylose | 0.848 | -0.286 |
|  | D-Mannitol | 0.869 | -0.086 |
|  | N-Acetyl-D-Glucosamine | 0.695 | -0.250 |
|  | D, L-α-Glycerol Phosphate | 0.287 | 0.440 |
| Amino acid | L-Asparagine | 0.813 | -0.232 |
|  | L-Phenylalanine | 0.806 | -0.042 |
|  | L-Arginine | 0.587 | -0.061 |
|  | L-Serine | 0.827 | -0.206 |
|  | L-Threonine | 0.757 | -0.075 |
|  | Glycyl-L-Glutamic Acid | 0.509 | 0.571 |
| Carboxylic Acid | γ-Hydroxybutyric Acid | 0.832 | -0.020 |
|  | Itaconic Acid | 0.781 | 0.114 |
|  | α-Ketobutyric Acid | 0.523 | 0.533 |
|  | D-Malic Acid | 0.275 | 0.260 |
|  | Pyruvic Acid Methyl Ester | 0.801 | -0.357 |
|  | D-Galacturonic Acid | 0.768 | -0.333 |
|  | D-Glucosaminic Acid | 0.764 | 0.046 |
| Polymers | α-Cyclodextrin | 0.737 | 0.268 |
|  | Glycogen | 0.737 | 0.426 |
|  | Tween 40 | 0.848 | -0.260 |
|  | Tween 80 | 0.862 | -0.279 |
| Amine | Phenylethylamine | 0.481 | 0.692 |
|  | Putrescine | 0.402 | 0.548 |
| Phenolic Acids | 2-Hydroxybenzoic Acid | 0.783 | 0.067 |
|  | 4-Hydroxybenzoic Acid | 0.858 | -0.012 |

Table S2 Biomarkers in different soil tillage

| Biomarker_names | Log_value | Groups | LDA_values | p_value |
| --- | --- | --- | --- | --- |
| k__Bacteria.p__Chloroflexi.c__Anaerolineae | 5.455 | T1 | 4.896 | 7.90884959353e-11 |
| k__Bacteria.p__Chloroflexi.c__Anaerolineae.o__Anaerolineales | 5.380 | T1 | 4.891 | 8.95042797974e-12 |
| k__Bacteria.p__Chloroflexi.c__Anaerolineae.o__Anaerolineales.f__Anaerolineaceae | 5.380 | T1 | 4.891 | 8.95042797974e-12 |
| k__Bacteria.p__Chloroflexi | 5.488 | T1 | 4.845 | 1.0557669444e-09 |
| k__Bacteria.p__Chloroflexi.c__Anaerolineae.o__Anaerolineales.f__Anaerolineaceae.g__UTCFX1 | 5.132 | T1 | 4.793 | 2.73765570659e-13 |
| k__Bacteria.p__Bacteroidota | 5.009 | T1 | 4.469 | 2.09372007293e-05 |
| k__Bacteria.p__Bacteroidota.c__Bacteroidia | 4.939 | T1 | 4.452 | 9.72394851622e-06 |
| k__Bacteria.p__Bacteroidota.c__Bacteroidia.o__Chitinophagales | 4.807 | T1 | 4.442 | 1.83132456476e-09 |
| k__Bacteria.p__Acidobacteriota | 5.175 | T1 | 4.428 | 1.22466240709e-05 |
| k__Bacteria.p__Bacteroidota.c__Bacteroidia.o__Chitinophagales.f__Chitinophagaceae | 4.685 | T1 | 4.317 | 2.27357848817e-09 |
| k__Bacteria.p__Acidobacteriota.c__Vicinamibacteria.o__Vicinamibacterales | 4.829 | T1 | 4.241 | 6.66466240097e-07 |
| k__Bacteria.p__Acidobacteriota.c__Blastocatellia | 4.436 | T1 | 4.110 | 2.73961221758e-12 |
| k__Bacteria.p__Acidobacteriota.c__Vicinamibacteria.o__Vicinamibacterales.f__Vicinamibacteraceae | 4.625 | T1 | 4.090 | 9.11779453041e-06 |
| k__Bacteria.p__Planctomycetota | 4.479 | T1 | 3.887 | 1.21383935301e-09 |
| k__Bacteria.p__Bacteroidota.c__Bacteroidia.o__Chitinophagales.f__Saprospiraceae | 4.192 | T1 | 3.839 | 5.94433051035e-07 |
| k__Bacteria.p__Bacteroidota.c__Bacteroidia.o__Chitinophagales.f__Chitinophagaceae.g__Flavisolibacter | 4.126 | T1 | 3.782 | 1.88122692309e-09 |
| k__Bacteria.p__Acidobacteriota.c__Blastocatellia.o__44524 | 4.078 | T1 | 3.754 | 8.34076981129e-12 |
| k__Bacteria.p__Myxococcota | 4.217 | T1 | 3.664 | 2.45687727186e-06 |
| k__Bacteria.p__Chloroflexi.c__Anaerolineae.o__Ardenticatenales | 4.006 | T1 | 3.644 | 3.02868161089e-13 |
| k__Bacteria.p__Acidobacteriota.c__Blastocatellia.o__Pyrinomonadales | 3.950 | T1 | 3.619 | 8.28509477747e-11 |
| k__Bacteria.p__Acidobacteriota.c__Blastocatellia.o__Pyrinomonadales.f__Pyrinomonadaceae.g__RB41 | 3.950 | T1 | 3.619 | 8.28509477747e-11 |
| k__Bacteria.p__Acidobacteriota.c__Blastocatellia.o__Pyrinomonadales.f__Pyrinomonadaceae | 3.950 | T1 | 3.619 | 8.28509477747e-11 |
| k__Bacteria.p__Acidobacteriota.c__Acidobacteriae | 4.169 | T1 | 3.607 | 0.000224010700953 |
| k__Bacteria.p__Planctomycetota.c__Phycisphaerae | 4.030 | T1 | 3.606 | 1.02348840561e-09 |
| k__Bacteria.p__Chloroflexi.c__Anaerolineae.o__Anaerolineales.f__Anaerolineaceae.g__Anaerolinea | 3.906 | T1 | 3.600 | 8.97774055845e-13 |
| k__Bacteria.p__Acidobacteriota.c__Acidobacteriae.o__Bryobacterales | 3.919 | T1 | 3.587 | 1.18975582792e-11 |
| k__Bacteria.p__Acidobacteriota.c__Acidobacteriae.o__Bryobacterales.f__Bryobacteraceae.g__Bryobacter | 3.919 | T1 | 3.587 | 1.18975582792e-11 |
| k__Bacteria.p__Acidobacteriota.c__Acidobacteriae.o__Bryobacterales.f__Bryobacteraceae | 3.919 | T1 | 3.587 | 1.18975582792e-11 |
| k__Bacteria.p__Armatimonadota | 3.994 | T1 | 3.569 | 6.41983595012e-08 |
| k__Bacteria.p__Bacteroidota.c__Ignavibacteria | 4.032 | T1 | 3.565 | 9.50131772226e-08 |
| k__Bacteria.p__Proteobacteria.c__Gammaproteobacteria.o__Burkholderiales.f__A21b | 3.866 | T1 | 3.563 | 2.80819154114e-11 |
| k__Bacteria.p__Proteobacteria.c__Gammaproteobacteria.o__Burkholderiales.f__SC_I_84 | 3.868 | T1 | 3.560 | 5.40963137103e-13 |
| k__Bacteria.p__Bacteroidota.c__Bacteroidia.o__Sphingobacteriales.f__AKYH767 | 3.872 | T1 | 3.532 | 1.36248672056e-09 |
| k__Bacteria.p__Acidobacteriota.c__Subgroup5 | 3.836 | T1 | 3.525 | 4.43400925517e-13 |
| k__Bacteria.p__Gemmatimonadota | 4.416 | T2 | 4.025 | 8.46080839281e-11 |
| k__Bacteria.p__Gemmatimonadota.c__Gemmatimonadetes.o__Gemmatimonadales | 4.341 | T2 | 3.954 | 5.57660669344e-10 |
| k__Bacteria.p__Gemmatimonadota.c__Gemmatimonadetes | 4.341 | T2 | 3.954 | 5.57660669344e-10 |
| k__Bacteria.p__Gemmatimonadota.c__Gemmatimonadetes.o__Gemmatimonadales.f__Gemmatimonadaceae | 4.341 | T2 | 3.954 | 5.57660669344e-10 |
| k__Bacteria.p__Proteobacteria.c__Alphaproteobacteria.o__Rhizobiales.f__Xanthobacteraceae | 4.434 | T2 | 3.900 | 1.0559423068e-07 |
| k__Bacteria.p__Proteobacteria.c__Gammaproteobacteria.o__Burkholderiales.f__B1_7BS | 4.189 | T2 | 3.852 | 3.88019890564e-11 |
| k__Bacteria.p__Latescibacterota | 4.173 | T2 | 3.780 | 1.18650806709e-10 |
| k__Bacteria.p__Proteobacteria.c__Gammaproteobacteria.o__Burkholderiales.f__Sutterellaceae | 4.020 | T2 | 3.694 | 5.48052208967e-10 |
| k__Bacteria.p__RCP2_54 | 4.002 | T2 | 3.656 | 3.4833633791e-14 |
| k__Bacteria.p__RCP2_54.c__metagenome | 3.879 | T2 | 3.560 | 5.44390490125e-14 |
| k__Bacteria.p__NB1_j | 3.834 | T2 | 3.510 | 5.00088392537e-09 |
| k__Bacteria.p__Sva0485 | 4.789 | T3 | 4.436 | 0.000229520826328 |
| k__Bacteria.p__Desulfobacterota.c__Desulfuromonadia.o__Geobacterales.f__Geobacteraceae.g__Trichlorobacter | 4.022 | T3 | 3.736 | 0.00010406069267 |
| k__Bacteria.p__Chloroflexi.c__Dehalococcoidia.o__S085 | 4.127 | T3 | 3.589 | 0.0096387771917 |
| k__Bacteria.p__Chloroflexi.c__Anaerolineae.o__ADurb_Bin180 | 3.867 | T3 | 3.539 | 1.15822539267e-09 |
| k__Bacteria.p__Proteobacteria.c__Gammaproteobacteria.o__Burkholderiales.f__Nitrosomonadaceae.g__mle1_7 | 3.878 | T3 | 3.537 | 1.90836939267e-09 |
| k__Bacteria.p__Chloroflexi.c__Anaerolineae.o__ADurb_Bin180.f__metagenome | 3.861 | T3 | 3.534 | 4.25002780898e-08 |
| k__Bacteria.p__Patescibacteria | 4.678 | T4 | 4.299 | 2.09745890891e-07 |
| k__Bacteria.p__MBNT15 | 4.452 | T4 | 3.999 | 0.00537247458105 |
| k__Bacteria.p__Proteobacteria.c__Gammaproteobacteria.o__Burkholderiales.f__Methylophilaceae.g__MM2 | 3.975 | T4 | 3.595 | 0.0350009219331 |
| k__Bacteria.p__Proteobacteria.c__Gammaproteobacteria | 5.523 | T5 | 4.996 | 3.50355368875e-11 |
| k__Bacteria.p__Proteobacteria | 5.576 | T5 | 4.980 | 1.99887088858e-10 |
| k__Bacteria.p__Proteobacteria.c__Gammaproteobacteria.o__Xanthomonadales.f__Xanthomonadaceae | 5.051 | T5 | 4.730 | 2.96507499851e-07 |
| k__Bacteria.p__Proteobacteria.c__Gammaproteobacteria.o__Xanthomonadales.f__Xanthomonadaceae.g__Lysobacter | 4.878 | T5 | 4.598 | 9.53519103756e-08 |
| k__Bacteria.p__Proteobacteria.c__Gammaproteobacteria.o__Burkholderiales.f__Oxalobacteraceae | 4.811 | T5 | 4.488 | 4.23623393071e-12 |
| k__Bacteria.p__Proteobacteria.c__Gammaproteobacteria.o__Burkholderiales.f__Oxalobacteraceae.g__Massilia | 4.793 | T5 | 4.471 | 5.04225438605e-12 |
| k__Bacteria.p__Chloroflexi.c__Dehalococcoidia | 4.519 | T5 | 4.130 | 9.01226419938e-08 |
| k__Bacteria.p__Actinobacteriota.c__Actinobacteria | 4.447 | T5 | 3.986 | 1.03780777009e-05 |
| k__Bacteria.p__Actinobacteriota.c__Actinobacteria.o__Micrococcales.f__Micrococcaceae | 4.257 | T5 | 3.924 | 1.03587565908e-12 |
| k__Bacteria.p__Actinobacteriota.c__Actinobacteria.o__Micrococcales.f__Micrococcaceae.g__Arthrobacter | 4.257 | T5 | 3.924 | 1.03587565908e-12 |
| k__Bacteria.p__Actinobacteriota.c__Actinobacteria.o__Micrococcales | 4.284 | T5 | 3.865 | 2.25608489747e-09 |
| k__Bacteria.p__Proteobacteria.c__Alphaproteobacteria.o__Caulobacterales.f__Caulobacteraceae | 3.939 | T5 | 3.599 | 1.47300286053e-10 |
| k__Bacteria.p__Proteobacteria.c__Alphaproteobacteria.o__Caulobacterales | 3.941 | T5 | 3.595 | 4.07334644657e-09 |
| k__Bacteria.p__Bacteroidota.c__Bacteroidia.o__Sphingobacteriales.f__Sphingobacteriaceae.g__Pedobacter | 3.845 | T5 | 3.581 | 9.14470693062e-07 |
| k__Bacteria.p__Methylomirabilota.c__Methylomirabilia.o__Methylomirabilales | 3.815 | T5 | 3.510 | 0.0027511414815 |
| k__Bacteria.p__Methylomirabilota.c__Methylomirabilia.o__Methylomirabilales.f__Methylomirabilaceae | 3.815 | T5 | 3.510 | 0.0027511414815 |

Table S3 Physicochemical properties of soil in different soil layers of tobacco-rice multiple cropping fields in Hunan

| Area | Tillage | SMC | SBD | SP | pH | SOC | AHN | TN | CNR |
| --- | --- | --- | --- | --- | --- | --- | --- | --- | --- |
|  |  | % | g/cm^3^ | % |  | g/kg | mg/kg | g/kg |  |
| Chenzhou | 0~10cm | 30.15 | 0.88 | 67.08 | 7.97 | 31.57 | 187.55 | 2.35 | 13.43 |
|  | 10~20cm | 28.13 | 1.05 | 60.97 | 8.03 | 29.36 | 175.43 | 2.23 | 13.17 |
|  | 20~30cm | 24.21 | 1.17 | 56.35 | 8.23 | 14.36 | 95.05 | 1.41 | 10.18 |
|  | 30~40cm | 19.87 | 1.47 | 45.29 | 8.23 | 7.76 | 37.64 | 0.95 | 8.16 |
|  | 40~50cm | 20.39 | 1.55 | 42.26 | 8.13 | 10.34 | 26.79 | 1.12 | 9.23 |
| Hengyang | 0~10cm | 24.24 | 1.09 | 57.09 | 5.17 | 16.71 | 138.43 | 1.61 | 10.38 |
|  | 10~20cm | 25.56 | 1.25 | 50.65 | 5.83 | 13.94 | 118.02 | 1.57 | 8.88 |
|  | 20~30cm | 21.09 | 1.47 | 42.24 | 6.03 | 6.77 | 59.33 | 1.01 | 6.71 |
|  | 30~40cm | 19.69 | 1.54 | 39.49 | 6.17 | 5.56 | 61.24 | 0.64 | 8.69 |
|  | 40~50cm | 19.15 | 1.53 | 39.88 | 6.27 | 5.02 | 77.19 | 0.75 | 6.70 |
| Changsha | 0~10cm | 19.17 | 1.29 | 47.66 | 7.80 | 14.93 | 120.57 | 1.49 | 10.02 |
|  | 10~20cm | 18.96 | 1.48 | 40.11 | 7.83 | 16.63 | 107.81 | 1.36 | 12.23 |
|  | 20~30cm | 15.20 | 1.61 | 34.70 | 7.90 | 10.06 | 61.24 | 1.16 | 8.67 |
|  | 30~40cm | 16.87 | 1.62 | 34.72 | 7.80 | 5.68 | 52.31 | 0.71 | 8.00 |
|  | 40~50cm | 17.71 | 1.62 | 34.55 | 7.77 | 2.82 | 45.93 | 0.35 | 8.05 |

Table S4 Redundancy analysis results of carbon source utilization ability and physicochemical properties of soil bacterial community

| physicochemical properties | RDA1 | RDA2 | r2 | Pr(>r) | Significance |
| --- | --- | --- | --- | --- | --- |
| SMC | 0.79420 | -0.60766 | 0.1464 | 0.037 | * |
| SBD | -0.99919 | -0.04022 | 0.2998 | 0.002 | ** |
| SP | 0.98992 | 0.14166 | 0.1968 | 0.010 | ** |
| pH | -0.70167 | 0.71250 | 0.2276 | 0.004 | ** |
| N | 0.99879 | 0.04909 | 0.2630 | 0.001 | *** |
| SOM | 0.96433 | 0.26469 | 0.2431 | 0.003 | ** |
| HN | 0.97168 | 0.23629 | 0.4571 | 0.001 | *** |
| C/N | 0.77804 | 0.62822 | 0.2758 | 0.002 | ** |

Note: ' ns ' means no significant difference, ' * ' means significant difference at the 0.05 level, ' * * ' means significant difference at the 0.01 level, ' * * * ' means significant difference at the 0.001 level.
